# Compound Specific Carbon Isotope Analysis in Sake by LC/IRMS and Brewers’ Alcohol Proportion

**DOI:** 10.1038/s41598-019-54162-6

**Published:** 2019-11-27

**Authors:** Momoka Suto, Hiroto Kawashima

**Affiliations:** Department of Management Science and Engineering, Faculty of Systems Science and Technology, Akita Prefectural University, 84−4, Ebinokuchi, Tuchiya, Yuri−Honjyo, Akita 015−0055 Japan

**Keywords:** Analytical chemistry, Organic chemistry, Characterization and analytical techniques, Mass spectrometry, Chemistry

## Abstract

Sake is a traditional Japanese alcohol. Nowadays, the consumption for Sake is increasing in worldwide and its popularity is growing. However, there are act of fraudulence by additional brewers’ alcohol and sugar. Therefore, a method is needed to find illegal fraud on label. In this work, we analyzed the δ^13^C values of the ethanol (δ^13^C_eth_) and glucose (δ^13^C_glu_) in Sake by liquid chromatography combined with isotope ratio mass spectrometry for the first time. Further, we developed the criteria using δ^13^C_eth_ and δ^13^C_glu_ to check brewers’ alcohol and sugar. In addition, there are some sake categories (Ginjyo and Futsu-shu) allowed to additional brewers’ alcohol up to legally determined percentage. The experimental additions of brewers’ alcohol from a C4 plant were conducted to Junmai, as sake by C3 plants. There was a strong correlation (*R* = 0.98, *P* < 0.05) between the percentage of added brewers’ alcohol and the δ^13^C values. We developed the method using the relationship for calculating percentage of brewers’ alcohol for the first time and estimated the percentage for commercial sake. Further, the price of sake was found to be inversely related to the percentage of brewers’ alcohol in the sake.

## Introduction

Sake, which is a traditional Japanese beverage made from rice, consists mainly of water, ethanol, carbohydrates, organic acids, and amino acids. It has been popular in Japan for a long time. According to the National Tax Agency of Japan^1^, there are 1415 sake breweries in Japan. The volume of sake sold in Japan was 534,494 kL in 2016. The export volume increased by a factor of 2.1 between 2008 and 2018 and was 25,747 kL in 2018. The main export destinations are the United States, South Korea, and China^2^.

Making sake is complicated by the fact that the starch in rice is a long polymer of sugar molecules that cannot be directly converted to alcohol by yeast. Rice starch is therefore broken down into sugar by enzymes produced by koji mold (saccharification). The sugar is then converted to alcohol by sake yeast (fermentation). The two functions of saccharification and fermentation proceed simultaneously (parallel double fermentation). Sake is specifically classified by the National Tax Agency of Japan. To simplify discussion, we classified sake into two initial categories: “Junmai”, which is made from only water, rice, and koji, and “Ginjo”, which is similar to Junmai but also includes brewers’ alcohol. Next, Junmai is further categorized based on the rice-polishing ratio. Junmai is categorized as “Junmai Daiginjo−shu”, “Junmai Ginjo−shu”, and “Junmai−shu” if the rice-polishing ratio is <50%, 50–60%, and 60–70%, respectively. The lower the rice-polishing ratio, the fruitier the flavor of the sake, and the higher the price. Ginjo is also categorized into three categories based on the rice-polishing ratio. It is categorized as “Daiginjo−shu”, “Ginjo−shu”, and “Honjozo−shu” if the rice-polishing ratio is <50%, 50–60%, and 60–70%, respectively. The inclusion of brewers’ alcohol in Ginjo has the effect of creating a lighter taste and increasing the aroma. Sake that does not fall into one of the above categories is classified as “Futsu−shu”. Brewers’ alcohol, sugars, and organic acids can be added to make Futsu−shu. The greatest quantity and the cheapest of the sake that is produced is Futsu−shu^1^. In all cases, the category of the sake is legally required to appear on the label.

On the one hand, the volume of Futsu−shu produced in Japan decreased by 19% from 2013 to 2017^1^. On the other hand, the corresponding volume of Junmai increased by 30%^1^. This difference is believed to reflect increased consumer demand for Junmai. The export volume of sake is also increasing, as mentioned above, and the fact that most of the exported sake is Junmai may reflect the difference in the tax rate of Junmai and Ginjo (Futsu−shu). There is an act of fraudulence by the price difference between the very popular kinds of Junmai sake and other kinds of sake. Recently, there have been some cases where Junmai was sold mixed with brewers’ alcohol.

Rice, which is the main ingredient in sake, is a C3 plant, and it has carbon stable isotope ratios (δ^13^C) that range from −30 to −22‰^3^. In contrast, the added brewers’ alcohol and sugar are produced mainly from C4 plants such as sugar cane, and they have δ^13^C values that range from −14 to −10‰^3^. Therefore, the δ^13^C of sake has proven to be an effective way to detect the addition of brewers’ alcohol^4–6^. In recent years, the δ^13^C values of various alcoholic beverages have been analyzed. For example, measurements have been made of the δ^13^C of wine^7–11^, beer^12,13^, sake^14–16^, brandies^17,18^, tequila^19^, liqueur^20^, whisky^21^, and shochu^16,22^. Bulk analyses of the δ^13^C of sake have produced values of −27.8‰^15^ and −20.6‰ (−20.9 to −20.3‰)^5^. However, it is difficult to detect the addition of sugar to sake by bulk analysis. Akamatsu, *et al*.^14^ have reported the δ^13^C value of the glucose (δ^13^C_glu_) in sake for the first time. After three cleanup steps using ion-exchange chromatography, they separated glucose using high-performance liquid chromatography. The glucose was then dried, and its δ^13^C was determined by elemental analyzer/isotope ratio mass spectrometry (EA/IRMS). They reported a difference in the δ^13^C_glu_ of sake with or without added sugar. However, this method requires a relatively large sample size (25 mL) and a long processing time (total about 20 h).

In the 2000s, liquid chromatography combined with isotope ratio mass spectrometry (LC/IRMS) was developed as an analytical tool. LC/IRMS is suitable for analyzing samples that contain polar and/or nonvolatile compounds. In addition, this method requires less sample volume and time because the analysis does not require sample preparation. LC/IRMS has recently been used to authenticate and trace products in various foods. The δ^13^C values have been reported using the flow-injection method in various alcoholic beverages (whiskey, brandy, vodka, tequila, and others). Analyses of individual compounds with LC/IRMS have included caffeine in beverages^23^; organic acids, glucose, and fructose in lemon juices^24,25^; glycerol and ethanol in wine^26–28^; vanillin in chocolate bars and snack foods^24^; xylitol in chewing gum^29^; and sugars (glucose, fructose, disaccharides, trisaccharides, and oligosaccharides) and organic acids in honey^30–35^. In this study, we collected 40 commercial sake samples and determined the δ^13^C values for the bulk sake (δ^13^C_bulk_), the ethanol (δ^13^C_eth_), and the glucose (δ^13^C_glu_) with LC/IRMS. In addition, we determined the corresponding δ^13^C values after adding brewers’ alcohol to Junmai−shu. Finally, we aimed to establish a quantitative method for calculating the proportion of brewers’ alcohol in commercial sake.

## Results and Discussion

### δ^13^C values in sake measured with LC/IRMS

Table 1 shows the δ^13^C values obtained for bulk sake, ethanol, glucose, and the differences between the components for 40 sake samples. For Junmai, previously reported δ^13^C values for δ^13^C_bulk_ in sake have been −27.36 ± 0.67‰^36^, −27.86 ± 0.22‰^16^, and −27.8‰^15^. The only previously reported δ^13^C_glu_ has been −26.1 ± 0.4‰^14^. Our values were close to these previously reported values and seemed to be reasonable. For δ^13^C_bulk_, δ^13^C_eth_, and δ^13^C_glu_, Junmai Daiginjo−shu, Junmai Ginjo−shu, and Junmai−shu were not significantly different from one another. Therefore, the rice-polishing ratio category could not be identified based on the δ^13^C results. Sasamoto *et al*.^36^ have reported similar results. The differences between the three components (δ^13^C_bulk_, δ^13^C_eth_, and δ^13^C_glu_) were very small. The ethanol and glucose in sake are produced by converting the starch in the same rice. The fact that the ethanol and glucose are produced from the same rice explains why there is little isotopic difference between them.

**Table 1:** δ^13^C of bulk sake, ethanol, and glucose, and paired differences of these three δ^13^C values.

Category	δ^13^C_bulk_	δ^13^C_eth_	δ^13^C_glu_	δ^13^C_bulk_ − δ^13^C_eth_	δ^13^C_bulk_ − δ^13^C_glu_	δ^13^C_eth_ − δ^13^C_glu_
Junmai (n = 15)	−28.2 ± 0.3	−28.3 ± 0.3	−27.7 ± 1.1	0.1 ± 0.2	−0.5 ± 1.0	−0.6 ± 1.0
Ginjo (n = 13)	−25.9 ± 0.3	−25.2 ± 0.6	−27.6 ± 0.8	−0.7 ± 0.3	1.7 ± 0.7	2.3 ± 0.6
Futsu-shu (n = 12)	−24.2 ± 1.8	−23.4 ± 2.0	−25.3 ± 4.0	−0.8 ± 0.5	1.0 ± 2.9	1.8 ± 3.4

In the case of Ginjo, the δ^13^C values for δ^13^C_bulk_ have previously been reported to be −25.09 ± 0.41‰^36^ and −24.08 ± 0.47‰^16^. These values are close to our results. The δ^13^C_bulk_ and δ^13^C_eth_ were 2.3 and 3.1‰ heavier, respectively, than the corresponding Junmai values. As mentioned above, brewers’ alcohol is produced mainly from C4 plants. The results of this study suggest that brewers’ alcohol from C4 plants was added to the sake. The fact that the δ^13^C_glu_ values for the Ginjo and Junmai were almost the same is reasonable, because the addition of sugar is illegal. The δ^13^C_bulk_, δ^13^C_eth_, and δ^13^C_glu_ values of the Daiginjo−shu, Ginjo−shu, and Honjozo−shu were not significantly different, as was the case with the Junmai δ^13^C values.

For Futsu-shu, the δ^13^C_bulk_ in this study was reasonable to comparison with −24.24 ± 0.88‰^36^, and lighter than the previously reported δ^13^C_bulk_ value of −20.81 ± 0.32‰^16^. The δ^13^C_bulk_, δ^13^C_eth_, and δ^13^C_glu_ were 4.0, 4.9, and 2.4‰ heavier, respectively, than the corresponding Junmai values. The fact that the Futsu-shu δ^13^C_eth_ was heavier than the Ginjo δ^13^C_eth_ may have been related to the amount of brewers’ alcohol added. The labels of samples 37, 38, 39, and 40 indicated that sugar had been added (Table S1). As shown above, not only brewers’ alcohol but also sugars and organic acids had been added to the Futsu−shu. For Futsu−shu, the δ^13^C_glu_ with sugar was −20.1 ± 2.0‰, which is 7.7‰ heavier than the δ^13^C_glu_ without sugar (−27.8 ± 1.0‰). Akamatsu, *et al*.^14^ have reported a δ^13^C_glu_ of −13.2 ± 3.3‰ for sake with sugar, which is 13.3‰ heavier than the δ^13^C_glu_ of −26.5 ± 0.4‰ for sake without sugar. The δ^13^C_glu_ is therefore a very effective assay for sugar addition.

### Scatter plot of the δ^13^C values of sake components

**Figure 1:**
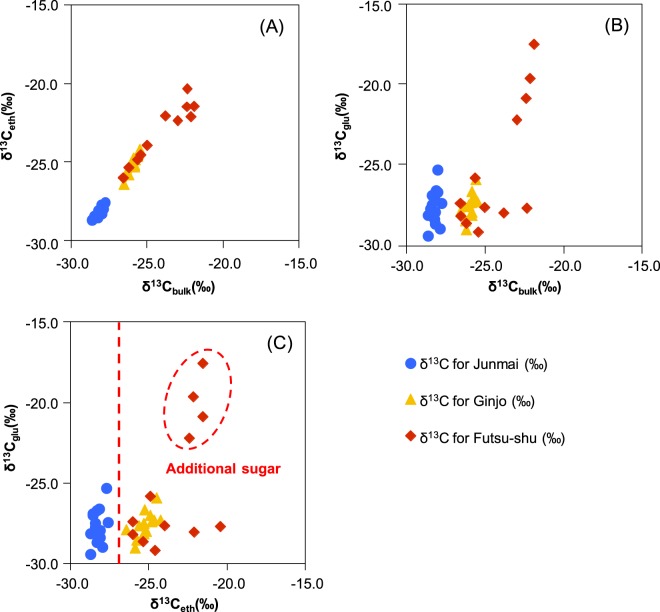
Scatter plots of δ13C of (**A**) bulk sake and ethanol, (**B**) bulk sake and glucose, and (**C**) ethanol and glucose in 40 sake samples.

Figure 1 shows a scatter plot of the δ^13^C values of the sake components. The fact that the δ^13^C_bulk_ and δ^13^C_eth_ were strongly correlated (*R* = 0.98, *P* < 0.05), as shown in Fig. 1(A), confirms that the principal carbon in sake is ethanol. Figure 1(B,C) show scatter plots between δ^13^C_bulk_ and δ^13^C_glu_, and between δ^13^C_eth_ and δ^13^C_glu_, respectively. If δ^13^C_eth_ exceeds −26.5‰, it is reasonable to conclude that brewers’ alcohol made from a C4 plant has been added to the sake. In addition, if δ^13^C_glu_ exceeds −23.4‰, it is reasonable to conclude that sugar has been added to the sake. The labels of the four samples within the solid circle in Fig. 1(C) indicated that sugar had been added to the sake (samples 37, 38, 39, and 40; Table S1). It is therefore reasonable to conclude that δ^13^C_eth_ and δ^13^C_glu_ are useful diagnostics for determining whether brewers’ alcohol and sugar, respectively, have been added to sake.

### Test of addition of brewers’ alcohol from a C4 plant

**Figure 2:**
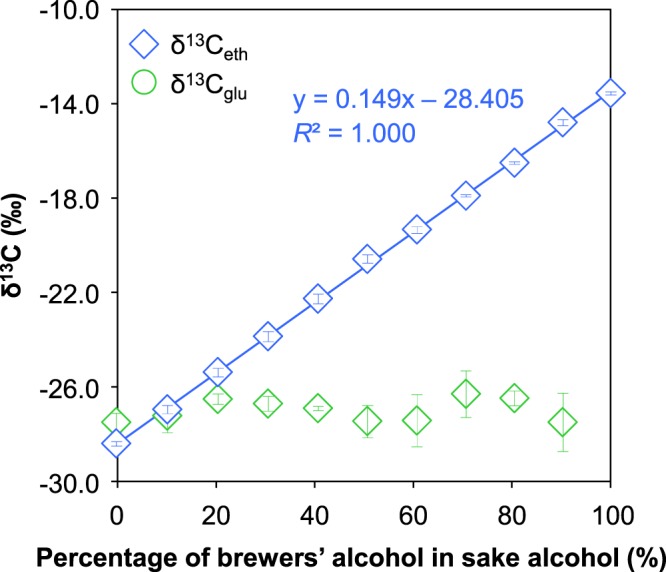
δ13C of ethanol and glucose versus percentage of brewers’ alcohol in sake alcohol.

The δ^13^C_eth_ of the three brewers’ alcohols available in this study were −13.6 ± 0.1‰, −13.8 ± 0.0‰, and −13.7 ± 0.1. These δ^13^C_eth_ values were very similar and strongly suggested that the brewers’ alcohol had come from C4 plants. One of the brewers’ alcohols was therefore used for the addition test (δ^13^C_eth_ = −13.6 ± 0.1‰, Oenon Holdings, Inc., alcohol content: 40%). Tests of additions of brewers’ alcohol from C4 plants were performed on Junmai−shu (sample 15) (Fig. 2). The δ^13^C_eth_ and δ^13^C_glu_ of that sample were found to be −28.4 ± 0.1‰ and −27.5 ± 0.4‰, respectively. As brewers’ alcohol is added, the δ^13^C_eth_ becomes heavier and approaches the δ^13^C_eth_ of brewers’ alcohol. There was a strong correlation (y = 0.149x  − 28.405, *R*^2^ = 1.000, where x is the percentage of added brewers’ alcohol and y is the δ^13^C_eth_ of the sake) between the percentage of added brewers’ alcohol and δ^13^C_eth_. A previous study has reported similar results (y = 0.159x − 27.812, *R*^2^ = 0.998)^16^. The δ^13^C_eth_ that results from the addition of brewers’ alcohol can be calculated as follows:

1 $${{\rm{\delta }}}^{13}{{\rm{C}}}_{eth}=\frac{({\delta }^{13}{C}_{eth-brew}\times brewers\,alcohol\,( \% ))+({\delta }^{13}{C}_{eth-Jun}\times (100-brewers\,alcohol( \% ))}{100}$$

where δ^13^C_eth-brew_ is the δ^13^C_eth_ (−13.6‰) of brewers’ alcohol, and δ^13^C_eth-Jun_ (−28.4‰) is the δ^13^C_eth_ of the Junmai−shu. The δ^13^C_eth_ values calculated from this equation and the measured values were very close (y = 0.148x −28.400, *R*^2^ = 1.000, where x is the percentage of added brewers’ alcohol and y is the δ^13^C_eth_ of the sake). The δ^13^C_glu_ value, however, did not change when brewers’ alcohol was added.

### Percentage of brewers’ alcohol in sake

**Figure 3:**
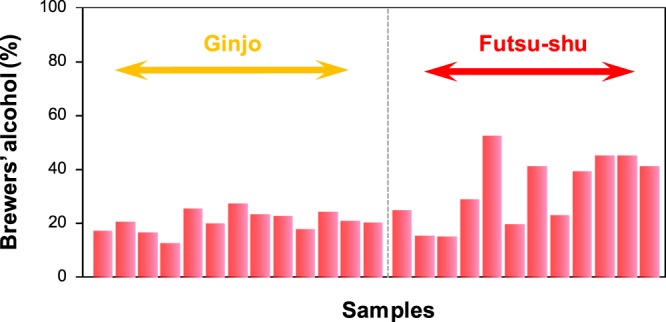
Percentage of brewers’ alcohol in Ginjo and Futsu-shu alcohol.

If brewers’ alcohol made from a C4 plant is added, the percentage of brewers’ alcohol in the Ginjo and Futsu−shu can be calculated using the equation y = 0.149 x − 28.405, where x is the percentage of added brewers’ alcohol and y is the δ^13^C_eth_ of the sake. For 25 sake samples, the percentages of brewers’ alcohol fell in the ranges 13.2–27.8% (mean: 21.3 ± 4.1%) for Ginjo and 15.7–53.5% (mean: 33.4 ± 13.1%) for Futsu−shu. Compared with Ginjo, the percentage of brewers’ alcohol in Futsu−shu covered a wider range (Fig. 3). Futsu−shu is allowed to contain brewers’ alcohol, sugar, amino acids, and organic acids until 50% by weight of the rice polishing, which is higher than the corresponding percentage for Ginjo (<10% by weight of the rice polishing). The wide range for the percentage of brewers’ alcohol in Futsu-shu was therefore likely a reflection of differences between breweries and products.

We estimated how much brewers’ alcohol was added by weight of the rice polishing in Ginjo. It is legal to add brewers’ alcohol to Ginjo at <10% by weight of the rice polishing. If 100 kg of rice polishing is used, at most 12.3 L (=10 kg) of brewers’ alcohol can be added. The results of this study indicate that brewers’ alcohol accounts for approximately 20% of the alcohol in Ginjo; the remaining 80% is derived from rice. In that case, the implication is that if 49.2 L of the alcohol in Ginjo produced from 100 kg of rice polishing, then another 12.3 L come from brewers’ alcohol. The National Research Institute of Brewing^37^ has reported that 194 L of Junmai (alcohol content: 18.3%) are produced from 100 kg of rice polishing. Thus, 35.5 L of alcohol are produced from 100 kg of rice polishing. The reported volume (35.5 L) of alcohol is lower than estimated volume (49.2 L) of alcohol produced from rice polishing. The reason was considered not to add until the legally maximum percentage (10%) of brewers’ alcohol. It was actually predicted that about 7%.

If the sugar added to a sake came from a C4 plant with a δ^13^C of −11‰^3^, the percentage of added sugar can be calculated from the δ^13^C_glu_ value of the sake and the equation y = 0.165x −27.500, *R*^2^ = 1.000, where x is the percentage of added sugar and y is the δ^13^C_glu_ of the sake. Our results indicate that the percentage of sugar added to samples 37, 38, 39, and 40 was 31.9, 60.3, 40.0, and 47.5%, respectively.

The price of the sake analyzed in this study was in the range 0.5–5.1 US$/100 mL (110 Japanese yen/US$). The mean prices of the Junmai and Ginjo were 2.5 ± 1.2 US$/100 mL and 2.0 ± 0.9 US$/100 mL, and there was no significant price difference between the Junmai and Ginjo. The mean price for Futsu−shu was lower: 0.9 ± 0.2 US$/100 mL. Futsu−shu cost about half as much as Ginjo and Junmai. The price of sake was proportional to approximately the inverse square of the percentage of brewers’ alcohol (*R*^2^ = 0.425) (Fig. S1). The weak correlation was probably caused by various differences in the costs of production, materials, and marketing at each brewery. The costs of Brazilian brandies have been shown to be lower for samples with relatively high percentages of added alcohol^18^. In this study, we calculated the percentages of brewers’ alcohol in Ginjo and Futsu−shu for the first time.

## Materials and Methods

### Reagents and samples preparation

Analytical standards of D−(+)−glucose (>98.0%) and ethanol (>99.5%) was purchased from Wako Pure Chemical Industries (Osaka, Japan). Sodium peroxodisulfate (Na_2_S_2_O_8_, >99.0%) was purchased from Sigma−Aldrich Co. (Tokyo, Japan), and phosphoric acid (H_3_PO_4_, >85.0%) was purchased from Kanto Chemical Co. (Tokyo, Japan). We purchased 40 kinds of sake at the local store. The Table S1 show category, prefecture of origin, alcohol content, price (US$/100 mL) and addition materials listed on the label. In this study, Junmai Daiginjo−shu, Junmai Ginjo−shu and Junmai−shu are referred as”Junmai”. In addition, Daiginjo−shu, Ginjo−shu and Honjozo−shu are referred as”Ginjo”. Sake without the above categories is classified as “Futsu−shu”. In this study, it was classified three categories as Junmai, Ginjo and Futsu−shu. (Table S1).

We purchased or provided three brewers’ alcohol samples from Oenon Holdings, Inc. (Tokyo, Japan), Kirin Holdings Company, Limited (Tokyo, Japan) and Takara Shuzo co., Ltd. (Kyoto, Japan). For δ^13^C analysis, the samples were diluted at 300−fold dilution for bulk, at 150−fold dilution for ethanol and 50−fold dilution for glucose in ultrapure water, respectively. All samples were filtered through 0.45 µm membrane filter (Sartorius Stedim Japan K.K., Minisart RC hydrophilic 17762Q).

### LC/IRMS instrument and δ^13^C calibration

**Figure 4:**
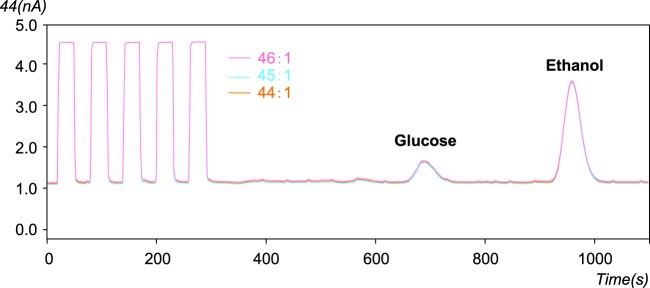
LC/IRMS chromatogram of ethanol and glucose in sake samples at 150-fold dilution.

The LC system (Shimadzu Co., Kyoto, Japan) was coupled to an IRMS instrument (Isoprime, Elementar UK, Manchester, UK) via a interface (LiquiFace, Elementar UK, Manchester, UK). The LC system consisted of three pumps (LC−10ADvp); column pump, post−column pump, and an oxidation pump, an autosampler (SIL−10ADvp), a degasser (DGU−14 A), a UV detector (SPD−10ADvp). The glucose and ethanol were separated on a Sugar−Pak I analytical column (300 mm × 6.5 mm, 10 μm particle size; Waters, Eschborn, Germany) at 80 °C. The eluent was ultrapure water (Milli−Q system, 18.2MΩ.cm; Millipore, Bedford, MA). The δ^13^C_bulk_ was analyzed using flow injection without column. The flow rates of column, post−column, and sodium peroxodisulfate + phosphoric acid were 0.5, 0.3 and 0.4 mL/min, respectively. All eluents were mixed and then degassed in an ultrasonic bath for 1 h. A pre−column filter (Chemicals Evaluation and Research Institute, Japan, Tokyo, Japan) was installed in front the column to prevent contamination. The injection volume was 10 μL for all analysis in this study. The system in this study was modified about the combustion heater, the cooling system, the gas separator, the helium flow meter passed on the outside of the membrane. The detail analytical method of the LC/IRMS in this research was previously described^38^. The IRMS instrument and the data acquisition system were controlled by a PC running Microsoft Windows XP Professional, and the LC was controlled by a PC running Windows 7 Ultimate. The IRMS and LC instruments were also controlled using IonVantage NT software (ver. 1.5.4.0., Isoprime) and LCsolution (ver. 1.25, Shimadzu Co., Kyoto, Japan), respectively. The analysis time for this study was approximately 18 minutes without pre−treatment (Fig. 4). For all samples, LC/IRMS data were measured in triplicate. To calibrate the system, five pulses of CO_2_ gas at the beginning of the run were conducted into the inlet of the mass spectrometer for approximately 30 s. The trap current was set at 300 μA. For LC/IRMS, δ^13^C values were normalized by international isotope standards of sucrose (IAEA−CH−6, δ^13^C = −10.449‰), and glucose (δ^13^C = −10.9‰), fructose (δ^13^C = −11.5‰), and galactose (δ^13^C = −27.8‰) obtained from EA/IRMS. The glucose, fructose, and galactose were normalized by international isotope standards of using the following international isotopic standards: IAEA−CH−3 (cellulose, δ^13^C = −24.724‰), IAEA−600 (caffeine, δ^13^C = −27.771‰), USGS24 (graphite, δ^13^C = −16.049‰), and IAEA−CH−6 (sucrose, δ^13^C = −10.449‰) using EA/IRMS. As a check of instrumental stability, an isotope working standard (ethanol, δ^13^C = −11.9‰, glucose, δ^13^C = −10.9‰) was analyzed after every 9 samples.

**Figure 5:**
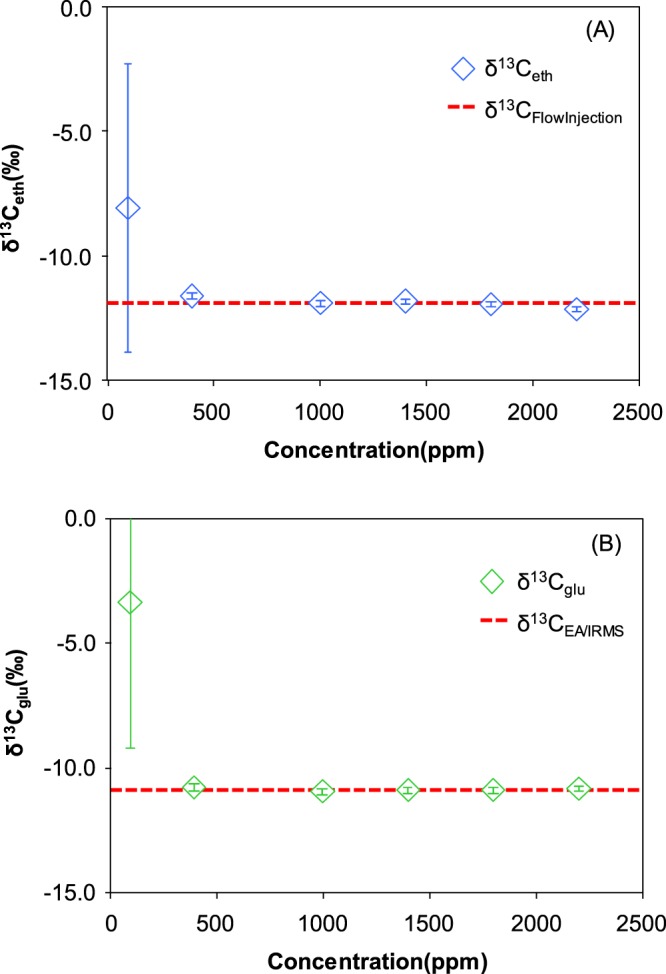
δ13C for (**A**) ethanol and (**B**) glucose at concentrations of 50–2000 ppm.

The relationships between the δ^13^C value and concentration between 100 and 2200 ppm are shown for ethanol and glucose in Fig. 5(A,B). At concentrations of 100 ppm, the accuracy and precision of the δ^13^C_eth_ and δ^13^C_glu_ were >3.8‰ (3.8‰ for ethanol and 7.6‰ for glucose) and >5.8‰ (7.0‰ for ethanol and 5.8‰ for glucose), respectively. At 400 to 2200 ppm, the accuracy was <0.3‰ for ethanol and glucose, and stable. The standard deviations of the ethanol and glucose were <0.4‰. Kawashima, *et al*.^38^ reported that the δ^13^C_glu_ were within ±0.5‰, >200 ppm, respectively. Therefore, the detection limit in this study seemed to be reasonable.

### Method of testing for addition of brewers’ alcohol

A test for addition of brewers’ alcohol from a C4 plant was performed on Junmai−shu. We used Junmai−shu (sample 15 in Table S1) and brewers’ alcohol (alcohol content: 44%) in this test. The 44% brewers’ alcohol was diluted to 16% brewers’ alcohol with ultrapure water. We mixed the Junmai−shu (sample 15) and 16% brewers’ alcohol to vary the brewers’ alcohol content in the sake over the range 10–90%. The sample mixtures were filtered through a 0.45-µm membrane filter and analyzed after being diluted as described above.

## Conclusions

We determined the δ^13^C_eth_ and δ^13^C_glu_ in 40 sake samples by LC/IRMS for the first time. The δ^13^C_eth_ and δ^13^C_glu_ ranged from −28.7 to −20.4‰ and −29.4 to −17.5‰, respectively. If the δ^13^C_eth_ and δ^13^C_glu_ values exceeded −26.5‰ and −23.4‰, brewers’ alcohol and sugar, respectively, were inferred to have been added to the sake. Tests of additions of brewers’ alcohol were performed on Junmai. As the percentage of added brewers’ alcohol increased, the δ^13^C_eth_ became heavier and approached that of brewers’ alcohol. There was a strong correlation (*R* = 0.98, *P* < 0.05) between the percentage of added brewers’ alcohol and the δ^13^C values. The percentage of brewers’ alcohol, which was calculated for the first time, fell in the range 13.2–53.5% for commercial sake. The price of sake was found to be inversely related to the percentage of brewers’ alcohol in the sake. It is therefore reasonable to conclude that δ^13^C_eth_ and δ^13^C_glu_ are useful diagnostics for determining whether brewers’ alcohol and sugar, respectively.

## Supplementary information

Supplementary information

## References

[1] National Tax Agency of Japan. *The production status of sake*, (in Japanese) (2017).

[2] National Tax Agency of Japan. *Overview of exports of sake manufacturers*, (in Japanese) (2017).

[3] Cerling TE (1997). Global vegetation change through the miocene/pliocene boundary. Nature.

[4] Schmidt HL (1986). Food quality control and studies on human nutrition by mass spectrometric and nuclear magnetic resonance isotope ratio determination. Fresenius’ J. Anal. Chem..

[5] Simpkins WA, Rigby D (1982). Detection of the illicit extension of potable spirituous liquors using ^13^C: ^12^C ratios. J. Sci. Food Agric..

[6] Rauschenbach P, Simon H (1979). Comparison of the deuterium and carbon-13 contents of ethanol obtained by fermentation and chemical synthesis. Z. Naturforsch., C: Biosci..

[7] Bréas O, Reniero F, Serrini G, Martin GJ, Rossmann A (1994). Isotope ratio mass spectrometry: Analysis of wines from different european countries. Rapid Commun. Mass Spectrom..

[8] Dordevic N (2013). Detecting the addition of sugar and water to wine. Aust. J. Grape Wine Res..

[9] Martinelli LA (2003). Stable carbon isotopic composition of the wine and CO_2_ bubbles of sparkling wines: Detecting C_4_ sugar additions. J. Agric. Food Chem..

[10] Raco B, Dotsika E, Poutoukis D, Battaglini R, Chantzi P (2015). O-H-C isotope ratio determination in wine in order to be used as a fingerprint of its regional origin. Food Chem..

[11] RoBmann A (1996). Stable carbon isotope content in ethanol of EC data bank wines from Italy, France and Germany. Z Lebensm Unters Forsch.

[12] Brooks JR (2002). Heavy and light beer: A carbon isotope approach to detect C _4_ carbon in beers of different origins, styles, and prices. J. Agric. Food Chem..

[13] Mardegan SF (2013). Stable carbon isotopic composition of Brazilian beers—A comparison between large- and small-scale breweries. J. Food Compos. Anal..

[14] Akamatsu F, Hashiguchi T, Igi Y, Izu H, Fujii T (2017). Carbon stable isotope analysis for glucose in sake: simple freeze-dried sake can substitute for glucose following HPLC isolation. Food Anal. Methods.

[15] Hashiguchi T, Akamatsu F, Izu H, Fujii T (2015). Preliminary detection method for added rice- and sugarcane-derived brewer’s alcohol in bulk samples of sake by measurement of hydrogen, oxygen, and carbon isotopes. Biosci. Biotechnol. Biochem..

[16] Horii S, Hashiguchi T, Igi Y, Izu H, Sudo S (2011). Speculation on the ratio of materials derived from C_4_ plants in Sake and Shochu. J. Brewing Soc. Jpn..

[17] Baudler R, Adam L, Rossmann A, Versini G, Engel KH (2006). Influence of the distillation step on the ratios of stable isotopes of ethanol in cherry brandies. J. Agric. Food Chem..

[18] Pissinatto L, Martinelli LA, Victoria RL, Camargo PBD (1999). Stable carbon isotopic analysis and the botanical origin of ethanol in Brazilian brandies. Food Res. Int..

[19] Bauer-Christoph C (2003). Authentication of tequila by gas chromatography and stable isotope ratio analyses. Eur. Food Res. Technol..

[20] Akamatsu F (2017). Method for the isolation of citric acid and malic acid in Japanese apricot liqueur for carbon stable isotope analysis. Food Chem..

[21] Parker IG, Kelly SD, Sharman M, Dennis MJ, Howie D (1998). Investigation into the use of carbon isotope ratios (^13^C/^12^C) of Scotch whisky congeners to establish brand authenticity using gas chromatography-combustion-isotope ratio mass spectrometry. Food Chem..

[22] Izu H, Hashiguchi T, Horii S, Sudo S, Matsumaru K (2012). Stable isotope analysis of commercially supplied *Honkaku Shochu*. Bunseki kagaku.

[23] Zhang L, Kujawinski DM, Federherr E, Schmidt TC, Jochmann MA (2012). Caffeine in your drink: natural or synthetic?. Anal.Chem..

[24] Bononi M, Quaglia G, Tateo F (2015). Easy extraction method to evaluate delta^13^C vanillin by liquid chromatography-isotopic ratio mass spectrometry in chocolate Bars and Chocolate Snack Foods. J. Agric. Food Chem..

[25] Guyon F (2014). ^13^C/^12^C isotope ratios of organic acids, glucose and fructose determined by HPLC-co-IRMS for lemon juices authenticity. Food Chem..

[26] Cabañero AI, Recio JL, Ruperez M (2010). Simultaneous stable carbon isotopic analysis of wine glycerol and ethanol by liquid chromatography coupled to isotope ratio mass spectrometry. J. Agric. Food Chem..

[27] Cabanero AI, Recio JL, Ruperez M (2008). Isotope ratio mass spectrometry coupled to liquid and gas chromatography for wine ethanol characterization. Rapid Commun. Mass Spectrom..

[28] Guyon F, Gaillard L, Salagoity MH, Medina B (2011). Intrinsic ratios of glucose, fructose, glycerol and ethanol ^13^C/^12^C isotopic ratio determined by HPLC-co-IRMS: toward determining constants for wine authentication. Anal. Bioanal. Chem..

[29] Koster D, Wolbert JB, Schulte MS, Jochmann MA, Schmidt TC (2018). Origin of xylitol in chewing gum: A compound-specific isotope technique for the differentiation of corn- and wood-based xylitol by LC-IRMS. J. Agric. Food Chem..

[30] Cabañero AI, Recio JL, Rupérez M (2006). Liquid chromatography coupled to isotope ratio mass spectrometry: A new perspective on honey adulteration detection. J. Agric. Food Chem..

[31] Dong H, Xiao K, Xian Y (2017). Isotope ratio mass spectrometry coupled to element analyzer and liquid chromatography to identify commercial honeys of various botanical types. Food Anal. Methods.

[32] Dong H, Xiao K, Xian Y, Wu Y (2018). Authenticity determination of honeys with non-extractable proteins by means of elemental analyzer (EA) and liquid chromatography (LC) coupled to isotope ratio mass spectroscopy (IRMS). Food Chem..

[33] Elflein L, Raezke KP (2008). Improved detection of honey adulteration by measuring differences between ^13^C/^12^C stable carbon isotope ratios of protein and sugar compounds with a combination of elemental analyzer - Isotope ratio mass spectrometry and liquid chromatography - Isotope ratio mass spectrometry (δ^13^C-EA/LC-IRMS). Apidologie.

[34] Kawashima H, Suto M, Suto N (2019). Stable carbon isotope ratios for organic acids in commercial honey samples. Food Chem..

[35] Suto, M., Kawashima, H. & Suto, N. Heart-cutting two-dimensional liquid chromatography combined with isotope ratio mass spectrometry for the determination of stable carbon isotope ratios of gluconic acid in honey. *J. Chromatogr. A*, 460421 (2019). 10.1016/j.chroma.2019.460421 31405574

[36] Sasamoto N, Abe Y, Takaku Y, Nakai I (2017). Characterization of japanese sake by trace-element compositions and isotope ratio of light elements. Bunseki kagaku.

[37] *Natinal Research Institute of brewing*, https://www.nrib.go.jp/ (2019).

[38] Kawashima H, Suto M, Suto N (2018). Determination of carbon isotope ratios for honey samples by means of a liquid chromatography/isotope ratio mass spectrometry system coupled with a post-column pump. Rapid Commun. Mass Spectrom..

