# Supplementary material for: Compound Specific Carbon Isotope Analysis in Sake by LC/IRMS and Brewers’ Alcohol Proportion
Source: Sci Rep. 2019 Nov 27;9:17635. doi: 10.1038/s41598-019-54162-6 (PMC6881313; doi:10.1038/s41598-019-54162-6)
Supplement: Supplementary file 1 — Supplementary information [file 41598_2019_54162_MOESM1_ESM.pdf]

Supplemental material

Compound Specific Carbon Isotope Analysis in Sake by LC/IRMS and Brewers' Alcohol Proportion

Momoka Suto and Hiroto Kawashima*

Department of Management Science and Engineering, Faculty of Systems Science and Technology, Akita Prefectural University, 84−4, Ebinokuchi, Tuchiya, Yuri−Honjyo, Akita, 015−0055, Japan

Corresponding Author:

*E-mail: kawashima@akita−pu.ac.jp

Table S1.

The category, prefecture of origin, alcohol content, price (US$/100mL), and additional information listed on the label of 40 sakes.

Figure S1.

Scatter plots of price versus the percentage of brewers’ alcohol in sake alcohol.

Table S1.

Sample	Category	Prefecture of origin	Alcohol content (%)	Price (US$/100 mL)	Additional information on label
1	Junmai Daiginjo-shu	Akita	16–17	4.5	–
2	Junmai Daiginjo-shu	Akita	15	1.5	–
3	Junmai Daiginjo-shu	Akita	16−17	3.5	–
4	Junmai Daiginjo-shu	Akita	15	3.4	–
5	Junmai Ginjo-shu	Akita	17	1.8	–
6	Junmai Ginjo-shu	Akita	15	2.9	–
7	Junmai Ginjo-shu	Akita	16	2.0	–
8	Junmai Ginjo-shu	Akita	15	2.1	–
9	Junmai Ginjo-shu	Akita	12−13	2.1	–
10	Junmai Ginjo-shu	Akita	16	2.2	–
11	Junmai Ginjo-shu	Yamagata	16	5.1	–
12	Junmai Ginjo-shu	Kyoto	15−16	1.3	–
13	Junmai-shu	Akita	16	1.5	–
14	Junmai-shu	Akita	16	1.8	–
15	Junmai-shu	Akita	15.5	1.4	–
16	Daiginjo-shu	Kyoto	15−16	1.4	brewers' alcohol
17	Daiginjo-shu	Akita	16−17	3.5	brewers' alcohol
18	Daiginjo-shu	Akita	16	3.3	brewers' alcohol
19	Daiginjo-shu	Ishikawa	16	3.3	brewers' alcohol
20	Ginjo-shu	Niigata	19	1.5	brewers' alcohol
21	Ginjo-shu	Fukushima	15	1.3	brewers' alcohol
22	Honjozo-shu	Kyoto	15−16	1.4	brewers' alcohol
23	Honjozo-shu	Niigata	19	1.3	brewers' alcohol
24	Honjozo-shu	Akita	14	1.4	brewers' alcohol
25	Honjozo-shu	Akita	15	2.0	brewers' alcohol
26	Honjozo-shu	Akita	14	2.3	brewers' alcohol
27	Honjozo-shu	Akita	15	1.3	brewers' alcohol
28	Honjozo-shu	Akita	15	1.5	brewers' alcohol
29	Futsu-shu	Akita	15	0.9	brewers' alcohol
30	Futsu-shu	Akita	15	1.0	brewers' alcohol
31	Futsu-shu	Akita	15	0.9	brewers' alcohol
32	Futsu-shu	Akita	15	1.0	brewers' alcohol
33	Futsu-shu	Akita	14−15	0.6	brewers' alcohol
34	Futsu-shu	Hyogo	19−20	1.3	brewers' alcohol
35	Futsu-shu	Hyogo	14−15	1.1	brewers' alcohol
36	Futsu-shu	Akita	14−15	1.2	brewers' alcohol
37	Futsu-shu	Akita	15−16	1.0	brewers' alcohol, sugar
38	Futsu-shu	Akita	15−16	1.0	brewers' alcohol, sugar, organic acids
39	Futsu-shu	Hyogo	13−14	0.7	brewers' alcohol, sugar, organic acids
40	Futsu-shu	Aichi	14	0.5	brewers' alcohol, sugar, organic acids

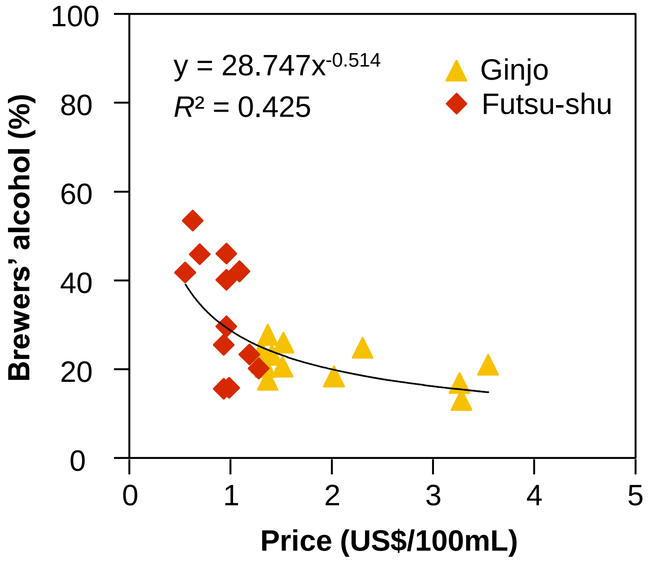
100
80
60
40
20
0
Brewers' alcohol (%)
0
1
2
3
4
5
Price (US$/100mL)
y = 28.747x-0.514
R² = 0.425
Ginjo
Futsu-shu

Figure S1.
